# Aberrant DNA methylation suppresses expression of estrogen receptor 1 (*ESR1*) in ovarian endometrioma

**DOI:** 10.1186/s13048-019-0489-1

**Published:** 2019-02-06

**Authors:** Ryo Maekawa, Yumiko Mihara, Shun Sato, Maki Okada, Isao Tamura, Masahiro Shinagawa, Yuichiro Shirafuta, Haruka Takagi, Toshiaki Taketani, Hiroshi Tamura, Norihiro Sugino

**Affiliations:** Department of Obstetrics and Gynecology, Yamaguchi University Graduate School of Medicine, Minamikogushi 1-1-1, Ube, 755-8505 Japan

**Keywords:** Endometriosis, Ovarian endometrioma, Eutopic endometrium, Steroid receptor, Estrogen receptor 1, Estrogen receptor 2, Progesterone receptor, DNA methylation

## Abstract

**Background:**

In ovarian endometriomas (OE), the expression statuses of various steroid hormone receptors are altered compared with their expression statuses in eutopic endometrium (EE). For example, in OE, the expressions of estrogen receptor 1 (*ESR1*)*,* which encodes ERα, and progesterone receptor (*PGR*) are downregulated, while the expression of *ESR2,* which encodes ERβ, is upregulated. The causes of these changes are unclear. DNA methylation of a specific region of a gene can result in tissue-specific gene expression. Such regions are called tissue-dependent and differentially methylated regions (T-DMRs). We previously reported that the tissue-specific expression of *ESR1* is regulated by DNA methylation of a T-DMR in normal tissues*.* In the present study, we examined whether aberrant DNA methylation of the T-DMR is associated with the altered expressions of *ESR1, ESR2 and PGR* in OE.

**Results:**

Gene expression levels of *ESR1, ESR2* and *PGR* were measured by quantitative RT-PCR. The expression levels of *ESR1* and *PGR* were significantly lower and the expression level of *ESR2* was significantly higher in OE than in EE. DNA methylation statuses were examined with an Infinium HumanMethylation450K BeadChip and sodium bisulfite sequencing. DNA methylation at the T-DMRs of *ESR1* were significantly higher in OE than in EE, but no significant differences were observed in the DNA methylation statuses of *ESR2* and *PGR*.

**Conclusions:**

Aberrant DNA methylation of the T-DMR was associated with the impaired expression of *ESR1*, but not the altered expressions of *ESR2* and *PGR*, in OE.

## Background

Endometriosis is a common gynecological disease affecting approximately 10% of reproductive age females [1], and is characterized by the ectopic localization of endometrial-like tissue in the pelvic cavity [1]. The disease induces chronic inflammation in the pelvic cavity, leading to symptoms such as chronic pelvic pain and infertility that subsequently affect the patient’s health [1, 2] .

Endometriosis develops mostly in women of reproductive age and regresses after menopause, suggesting that the growth is estrogen-dependent. Estrogen hormone action is mediated by the estrogen receptor in many physiological and pathological processes. The estrogen receptor has two subtypes, estrogen receptors α and β (ERα and ERβ) encoded by estrogen receptor 1 (*ESR1*) and 2 (*ESR2*), respectively [3, 4]. *ESR1* is the primary mediator of the estrogenic action, and its expression of *ESR1* is higher than that of *ESR2* in endometrium [5]. In endometriotic tissue, the expression of *ESR1* is strongly suppressed, while *ESR2* is upregulated [6]. However, the exact mechanisms of the downregulation of *ESR1* and upregulation of *ESR2* in endometriotic tissue remain unclear [7, 8].

DNA methylation is a well-known epigenetic mark. DNA methylation, which occurs at CpG sites, interrupts the recognition and binding of transcription factors [9], recruits methyl CpG-binding proteins that interact with transcription repressors [9], and induces chromatin condensation via histone modification changes [9]. Maintaining a specific DNA methylation profile in a cell is necessary for cellular integrity, and alterations in DNA methylation may have serious health consequences [9]. We and other groups have previously demonstrated that aberrant epigenetic regulation is associated with the pathogenesis and development of endometriosis [10–13] . In fact, local estrogen production is upregulated in endometriotic tissue by altering the activities of enzymes involved in synthesizing or degrading estradiol [10, 14, 15]. We showed that the altered activities of these genes were caused by aberrant DNA methylation [10, 16].

The tissue- and cell-specific expression of a gene can be determined by DNA methylation of a specific region of the gene called the tissue-dependent and differentially methylated region (T-DMR) [17]. Recent genome-wide analyses have identified many T-DMRs in mammalian genomes [17–19]. T-DMRs tend to be located at an upstream region of gene promoter or intron rather than in the gene promoter region [18–21]. We previously reported that two T-DMRs are present in the region from − 0.5 kb to − 1 kb with respect to the transcription start site of *ESR1*, and DNA methylation of the two T-DMRs, but not the promoter region, regulate tissue-specific expression of *ESR1* in normal tissues [20]. In the tissues with DNA hypermethylation in the two T-DMRs, the expression of *ESR1* is suppressed [20]. This led us to hypothesize that the downregulation of *ESR1* in endometriosis is caused by aberrant DNA methylation of these T-DMRs. On the other hand, DNA methylation of the promoter region (not the T-DMRs) was reported to regulate the expression of *ESR2* [22]. However, because the T-DMRs have a role in *ESR1* expression, there is a possibility that regions other than the promoter region are involved in the regulation of *ESR2* expression.

Resistance to progesterone may contribute to the pathophysiology of endometriosis [23], and may be caused by aberrant expression of progesterone receptor (*PGR*) [24]. In cancer tissues, *PGR* expression may be regulated by DNA methylation of the proximal promoter region of *PGR* [25, 26]. However, it remains unclear whether aberrant DNA methylation is also involved in the suppressed expression of *PGR* in endometriosis.

In the present study, we first compared the expressions of *ESR1*, *ESR2 and PGR* in eutopic endometrium (EE) and ovarian endometrioma (OE). Next, we examined the DNA methylation statuses of *ESR1*, *ESR2* and *PGR* to investigate whether aberrant DNA methylation is associated with their aberrant expressions in OE.

## Results

### mRNA expressions of ESR1, ESR2 and PGR

**Fig. 1:**
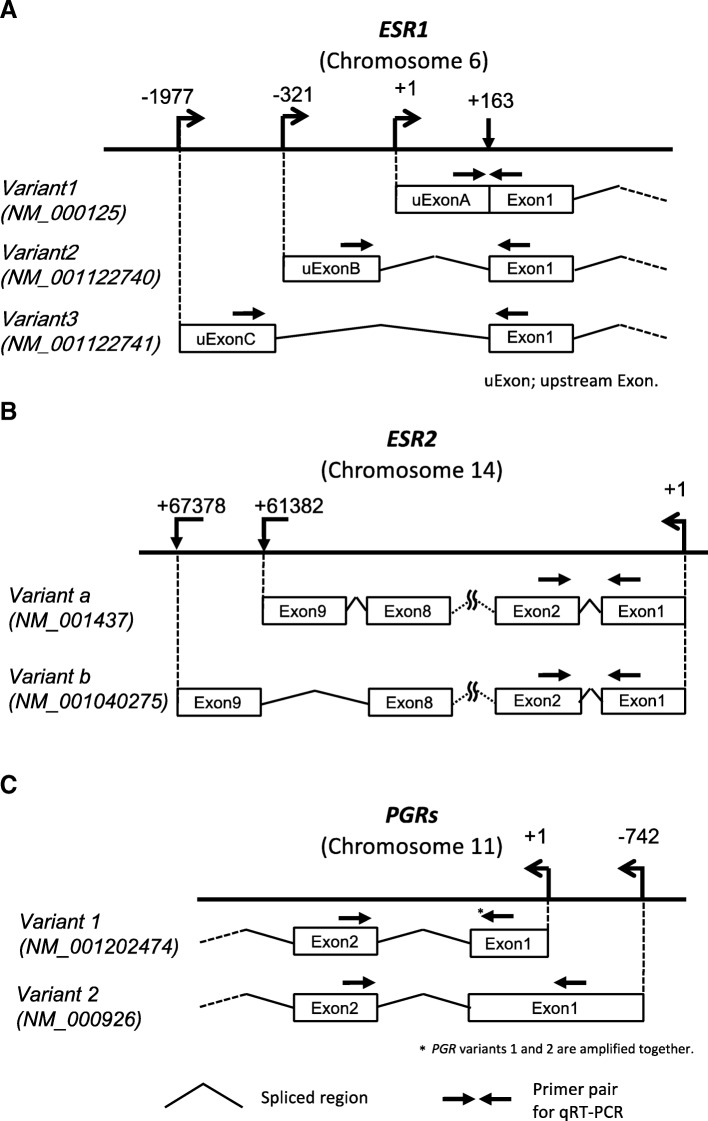
Genomic organization of *ESR1*, *ESR2* and *PGR*. **a** Upstream exons and corresponding transcription start sites (TSSs) of *ESR1*. The upstream exons are shown by boxes and the corresponding TSSs are indicated by arrows. The numbers show the positions of the 5′ start sites of the upstream exons with respect to the start site of upstream Exon A. The different start sites correspond to the mature *ESR1* mRNAs, called variant 1, 2 and 3. All 5′ upstream exons are spliced at the common acceptor splice site (+ 163 bp). **b** Exons, transcription start sites and transcription end sites of *ESR2*-variant a and b. The numbers of Exon 9 indicate the distances from the transcription start sites of each variant. **c** Exons, transcription start sites of *PGR*-variant 1 and 2. The numbers associated with Exon 1 of variants 1 and 2 indicate the 5′ start sites with respect to the distance from the start site of variant 1 (+ 1). The locations of the primer pairs used in qRT-PCR are indicated by the arrows

**Fig. 2:**
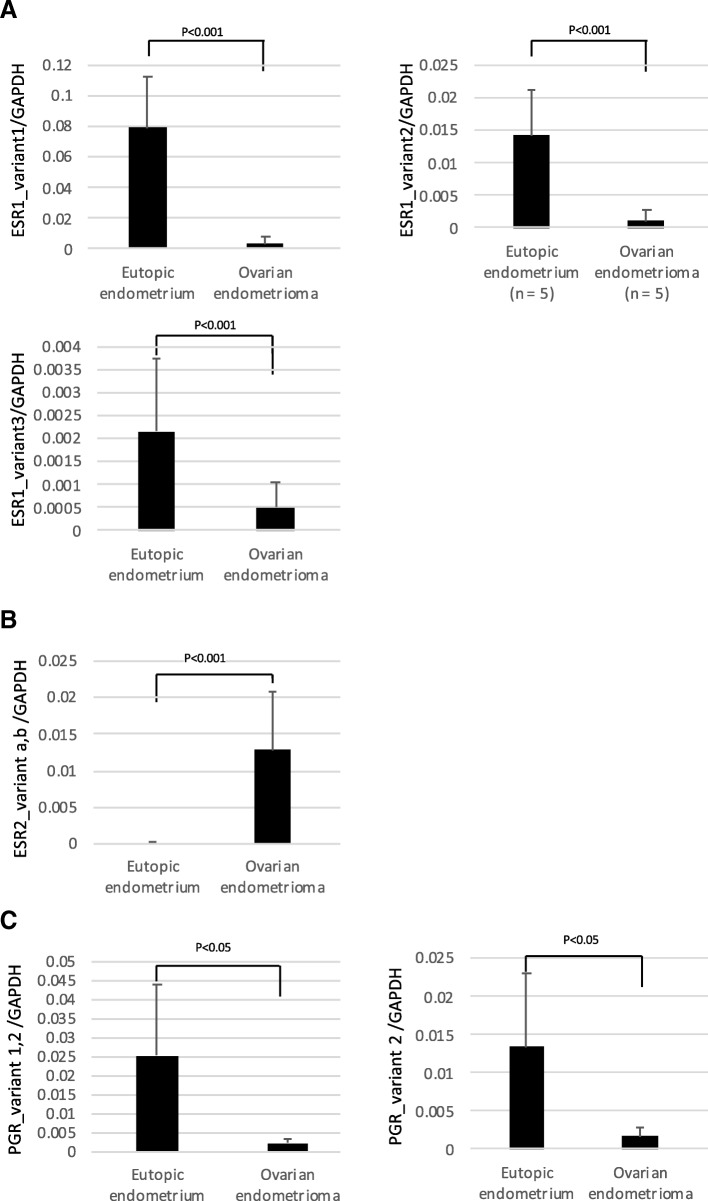
mRNA expression statuses of *ESR1*, *ESR2* and *PGR* variants in EE and OE. *ESR1* (variant 1, 2, and 3) (**a**) *ESR2* (variant a and b) (**b**) and *PGR* (variant 1 and 2) (**c**) were analyzed by qRT-PCR. GAPDH was used as an internal control. The amount of mRNA of each variant was normalized to that of the internal control (*GADPH*). Data were expressed as a ratio of mRNA of each variant to *GADPH*. Each bar represents the mean +/− SEM.* *p* < 0.05

*ESR1* has two types of exons: four non-coding upstream-exons (uExons), the most common of which are uExons A, B and C [27], in addition to its coding exons (Exons 1, 2, 3...). The *ESR1* mRNA has only one uExon spliced to the coding exons, so that the transcription start site of *ESR1* depends on which uExon is used (Fig. 1a) [27]. The mRNA variants corresponding to uExons A, B and C are called variants 1, 2 and 3, respectively. Because the translation start site (ATG) is located on Exon 1, the uExon does not affect the protein product of *ESR1*. The mRNA expression statuses of variants 1, 2 and 3 were analyzed in EE and OE by qRT-PCR. Their transcript levels were significantly higher (23.3-, 12.6- and 4.4-folds, respectively) in EE than in OE (Fig. 2a).

*ESR2* has two isoforms encoded by *ESR2* variants a and b (Fig. 1b). The expression levels of the two variants were examined together using a primer pair that detects both variants. The expression of *ESR2* was 134-fold higher in OE than in EE (Fig. 2b).

*PGR* has two isoforms, *PR-A* and *PR-B*, encoded by *PGR* variants 1 and 2, respectively (Fig. 1c). Since the primer pair designed for *PGR* variant 1 also detects *PGR* variant 2, the amplified product reflects the combined levels of *PGR* variants 1 and 2. The combined expression level of *PGR* variants 1 and 2 was 12-fold lower in OE than in EE (Fig. 2c). The expression of *PGR* variant 2 was 8-fold lower in OE than in EE (Fig. 2c).

### DNA methylation statuses detected by 450K BeadChip

**Fig. 3:**
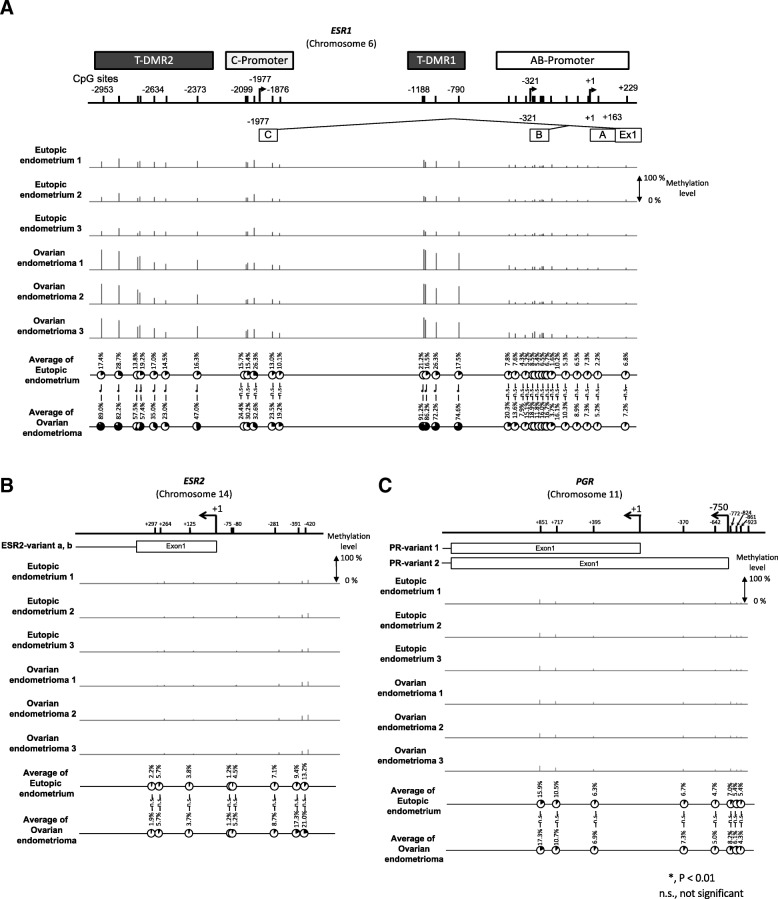
DNA methylation statuses of *ESR1*, *ESR2* and *PGR* in EE and OE samples by BeadChip. Samples from 3 subjects are shown for each sample type. **a** DNA methylation statuses of AB-promoter, T-DMR1, C-promoter, and T-DMR2. The locations of each upstream Exon and CpG site are shown with the distance from the TSS of upstream Exon A. A, B and C are upstream Exon A, B and C, respectively. **b** and **c** DNA methylation statuses of the region of the transcription start site of *ESR2* (**b**) *and PGR* (**c**). Bars indicate methylation levels from 0 to 100%. Pie charts show the average DNA methylation statuses (percentage) of EE and OE at each CpG site. The DNA methylation statuses of each CpG site were compared by unpaired *t*-test. *p* < 0.05

*ESR1* has two T-DMRs (T-DMR1 and T-DMR2), upstream of the AB-promoter and upstream of the C-promoter, respectively (Fig. 3a) [20]. We first examined the DNA methylation status of the region from − 2953 bp to + 229 bp of *ESR1* with the 450 K BeadChip. DNA methylation was low in the AB-promoter in both EE and OE (Fig. 3a). The DNA methylation statuses of these tissues were not significantly different. On the other hand, the DNA methylation status of T-DMR1 was significantly lower in EE than in OE (*p* < 0.05, Fig. 3a). Similarly, the DNA methylation status in the C-promoter was low in EE and OE, while the DNA methylation status in T-DMR2 was low in EE and high in OE (Fig. 3a). The DNA methylation status of T-DMR2 was significantly different between EE and OE (*p* < 0.05, Fig. 3a). The DNA methylation status of *ESR2* was low in EE and OE and the levels were not significantly different (Fig. 3b). The DNA methylation statuses of *PGR*-variants 1 and 2 were low and not significantly different in EE and OE (Fig. 3c).

### DNA methylation statuses detected by sodium bisulfite sequencing

**Fig. 4:**
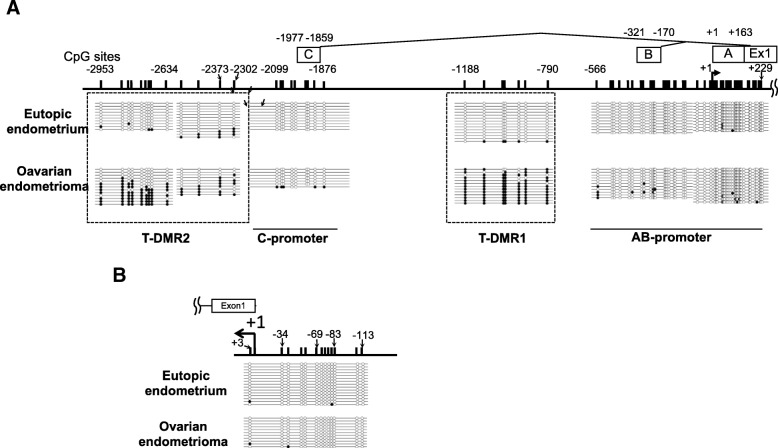
DNA methylation statuses of *ESR1* and *ESR2* in EE and OE by sodium bisulfite sequencing. One sample was analyzed for each sample type. For each region, 7 to 15 clones were sequenced. The methylation status of each CpG site in each clone is shown as unmethylated (open circles) or methylated (closed circles). **a** The numbers associated with uExons and CpG sites indicate the distance from transcription start site (TSS) of uExon A. A, B, and C indicate uExons A, B, and C, respectively. **b** The numbers associated with CpG sites indicate the distance from TSS of Exon 1

In *ESR1*, DNA methylation levels of the AB- and C-promoters were low in both EE and OE (Fig. 4a). On the other hand, the DNA methylation levels of T-DMR1 and T-DMR2 were low in EE and high in OE (Fig. 4a). In *ESR2*, DNA methylation was low in both EE and OE (Fig. 4b).

## Discussion

We previously reported that DNA methylation of the T-DMRs of *ESR1* regulates the expression of *ESR1* in normal tissues [20]. The expression of *ESR1* in the tissues with high DNA methylation at the T-DMRs was strongly suppressed compared with the expression of *ESR1* in the tissues with low DNA methylation at the T-DMRs [20]. In the present study, *ESR1* expression was lower and DNA methylation at the T-DMRs of *ESR1* was higher in OE than in EE, indicating that the aberrant DNA methylation of the T-DMRs was associated with the impaired expression of *ESR1* in OE. On the other hand, DNA methylation was not involved in the altered expressions of *ESR2* and *PGR* in OE.

*ESR1* is downregulated in cancer tissues such as breast cancers, and the downregulation was shown to be associated with DNA methylation of the promoter region of *ESR1* [28–30]. However, Meyer et al. [7] found no significant difference in the DNA methylation status of *ESR1* at the promoter region between EE and OE. We previously showed that methylation of the promoter region (not the T-DMR) of *ESR1* was low in normal tissues with both high and low *ESR1* expression [20, 31]. These results indicated that the DNA methylation of the T-DMRs regulates the expression of *ESR1* and there is no difference in DNA methylation of the promoter regions between tissues with different expression levels of *ESR1* [20]. The present results show that the methylation levels of the promoter regions were kept low in both OE and EE, which indicates that the impaired expression of *ESR1* in OE is due to the T-DMRs and not the promoter region.

*ESR2*, which is upregulated in OE, has been shown to suppress *ESR1* expression in endometriosis by binding to the *ESR1* promoter [8, 32] . Knockdown of *ESR2* in endometriotic stromal cells increased the expression of *ESR1* twofold, and overexpression of *ESR2* in endometrial stromal cells reduced the expression of *ESR1* by one-half [8, 32]. In view of the huge difference in the expression levels of *ESR1* between EE and OE (Fig. 2a), aberrant DNA methylation of the T-DMRs is likely a factor in the impaired expression of *ESR1* in OE.

Xue et al. reported that the DNA methylation levels of the promoter region of *ESR2* is high in endometrial stroma, while it is low in endometriotic stroma [22]. They concluded that the increased expression of *ESR2* in endometriosis is caused by DNA hypomethylation of the promoter region (from − 163 to − 48) of *ESR2* [22, 32]. However, 25% of their endometrial stroma samples also showed DNA hypomethylation. Because of this inconsistency, they may have shown only a difference among individuals. The present results show that DNA methylation in the region from − 163 to − 48 was low in both EE and OE. We also did not see any difference in DNA methylation in the regions upstream and downstream of this region; in these regions, both EE and OE were DNA hypomethylated. Our results suggest that DNA methylation is not involved in the upregulation of *ESR2* in OE.

The expressions of *PGR* variants 1 and *2* were significantly different between EE and OE. The present study showed no significant difference in the DNA methylation status between EE and OE, indicating that DNA methylation is not associated with the differential expression of *PGR*. However, in breast cancer tissues, DNA methylation around the transcription start sites appears to have a role in the regulation of *PGR* expression [25, 26]. Aberrant DNA methylation often occurs in the promoter region in a variety of cancers [33, 34]. In fact, we previously showed that DNA hypermethylation of the *ESR1* promoter occurred only in cancer tissues and that this region remained hypomethylated in non-cancer tissues regardless of the *ESR1* expression level [20]. Therefore, the difference between the previous reports and the present study may be due to a difference in the examined samples. Other mechanisms such as the binding of transcription factors may be associated with the differential expression of *PGR* between EE and OE.

## Conclusions

We found that OE had aberrant DNA methylation in the T-DMRs of *ESR1* and that the DNA methylation is associated with the impaired expression of *ESR1* in OE. On the other hand, it is unlikely that DNA methylation is associated with the altered expressions of *ESR2* and *PGR* in OE.

## Methods

### Tissue samples

Specimens of EE and OE were obtained from 16 Japanese women. EE was obtained from patients who underwent surgery for uterine leiomyoma. The ages of the patients were 32.13 +/− 4.19 years old (mean +/− SD; 26–40 years). OE tissue was obtained from patients who underwent cystectomy of OE during the proliferative phase [10, 16]. The ages of the patients were 31.13 +/− 5.08 years old (mean +/− SD; 24–39 years old) and did not significantly differ from the patients with uterine leiomyoma (*p* = 0.67). None of the patients had received previous hormonal therapy. Specimens were dissected immediately after removal, immersed in liquid nitrogen and stored at − 80 °C until DNA/RNA extraction as previously reported [35, 36].

### Quantitative real-time RT-PCR (qRT-PCR)

Total RNA was isolated from tissues using Isogen (Wako Pure Chemical Industries Ltd., Osaka, Japan). One 1 μg total RNA was reverse-transcribed using a Quantitect Reverse Transcription Kit (Qiagen, Valencia, CA, USA) according to the manufacturer’s protocol as previously reported [37]. To distinguish the transcribed variants derived from each upstream Exon (Fig. 1a), we synthesized three primer pairs as shown in Fig. 1a and Table 1. *ESR2* is also known to have two major variants, variant a and b (Fig. 1b). We made a primer pair that amplifies both variants (Fig. 1b and Table 1). *PGR* has two isoforms, *PR-A* and *PR-B*, encoded by *PGR*-variants 1 and 2, respectively (Fig. 1c). Since all nucleotides in *PGR*-variant 1 are included in *PGR*-variant 2, the primer pair designed for *PGR* variant 1 also amplified *PGR* variant 2. We designed two primer pairs. One detected the combined expression level of *PGR* variants 1 and 2, and the other detected *PGR*-variant 2 specifically (Fig. 1c and Table 1). A primer pair for glyceraldehyde-3-phosphate dehydrogenase (*GAPDH*) was used as an internal control (Table 1). Real-time qRT-PCR was performed with 5 specimens each of EE and OE using SYBR Premix Ex Taq (Takara, Ohtsu, Japan) and a LightCycler (Roche Applied Science, Basel, Switzerland). All samples were run in duplicate. The relative quantity of cDNA was calculated with the ∆∆Ct method. Melting curves of the products were obtained after cycling by a stepwise increase of temperature from 55 to 95 °C.

**Table 1:** Primer sets used in quantitative RT-PCR

Name	Forward
ESR1	
all variants	F: 5′-TGTGCAATGACTATGCTTCA-3′
variant1 (transcribed from uExon-A)	F: 5′-CTCGGGCTGTGCTCTTTTT-3′
variant2 (transcribed from uExon-B)	F: 5’-GCCGTGAAACTCAGCCTCTA-3′
variant3 (transcribed from uExon-C)	F: 5′-TGGAACATTTCTGGAAAGACG-3′
ESR2	
variant-a/variant-b	F: 5′ -CTCGCTTTCCTCAACAGGTG- 3′
PGR	
variant1/variant2	F: 5′ -ACCAGCTCTTGGTGCCTGT- 3′
variant2	F: 5′ -TCCCTCTGCCCCTATATTCC- 3′
GAPDH	F: 5′-AGGTGAAGGTCGGAGTCA-3′

### Illumina Infinium HumanMethylation450k BeadChip assay

Genomic DNA was isolated from 20 mg of frozen tissue using a Qiagen Genomic DNA kit (Qiagen). DNA methylation was analyzed with an Illumina Infinium assay with the HumanMethylation450 BeadChip (Illumina, San Diego, CA, USA), which interrogates a total of 482,421 CpG sites from the distal promoter regions of the transcription start sites to the 3’-UTR of consensus coding sequences. Methylated and unmethylated signals were used to compute beta values, which are quantitative scores of the DNA methylation levels, ranging from 0 (completely unmethylated) to 1 (completely methylated). The BeadChip was scanned on a BeadArray Reader (Illumina) according to the manufacturer’s instructions. CpG sites with “detection p values” > 0.05 (computed from the background based on negative controls) and CpG sites on the Y chromosome were eliminated from further analysis, leaving 482,005 CpGs valid for use with the nine samples tested.

### Sodium bisulfite sequencing

Bisulfite reactions were performed using an EpiTect Bisulfite Kit (Qiagen) with the following temperature conditions: 95 °C for 5 min, 65 °C for 85 min, 95 °C for 5 min and 65 °C for 175 min as previously reported [38]. The bisulfite-converted DNA was amplified by PCR using the primer pairs for *ESR1* and *ESR2* shown in Table 2 using the following thermocycling conditions: 95 °C for 10 min, and 40 cycles of 94 °C for 30 s, 60 °C for 30 s and 72 °C for 1 min followed by 10 min of final extension at 72 °C. The resulting products were cloned into a pGEM-T easy vector (Promega, Tokyo, Japan). The vectors were sequenced using a BigDye Terminator V3.1 Kit (Applied Biosystems, Foster City, CA, USA) and a 3130xl Genetic Analyzer (Applied Biosystems) as previously reported [20]. QUMA (http://quma.cdb.riken.jp/) was used to analyze the bisulfite sequencing data [39].

**Table 2:** Primer sets used in sodium bisulfite sequencing analysis

Name	Forward	Reverse
ESR1_Region I (− 129 to + 279)	F: 5′-GTTGTGTTTGGAGTGATGTTTAAGTT-3′	R: 5′-CAATAAAACCATCCCAAATACTTTA-3′
ESR1_Region II (− 670 to −94)	F: 5′-GGAAGGGTTTATTTATTTTGGGAGTA-3′	R: 5′-TAACATTAACTTAAACATCACTCC-3′
ESR1_Region III (− 1298 to − 731)	F: 5′-TTGGGTGTTTGGGATAGTAATTAAA-3′	R: 5′-CTTAATCCCATTAAAAATTCTCAT-3′
ESR1_Region IV (− 2227 to − 1756)	F: 5′-TAGTTTTTAAGGGTAGGGGTAAAGG-3′	R: 5′-CAACAATCCTCATCTCCCTACTAAA-3′
ESR1_Region V (− 2595 to − 2241)	F: 5′-TATTTATGGAAAGGTTTGTGGGTTT-3′	R: 5′-TACTTTCTACTACCACCCCAAACAA-3′
ESR1_Region VI (− 2981 to − 2573)	F: 5′-AATTGTATAGTGTTTTAGGGTTAGAGA-3′	R: 5’-ACCCACAAACCTTTCCATAAATAAC-3’
ESR2 (− 113 to + 3)	F: 5′-ATTATTTTTGTGGGTGGATTAGGAG-3′	R: 5′-AACCCCTTCTTCCTTTTAAAAACC-3′

### Statistical analyses

DNA methylation and mRNA Expression levels of the two groups were compared with unpaired *t*-tests using SPSS for Windows version 11 (SPSS Inc., Chicago, IL, USA).

## Abbreviations

EE: Eutopic endometrium
ERα: Estrogen receptor alpha
ERβ: Estrogen receptor beta
*ESR1*: Estrogen receptor 1
*ESR2*: Estrogen receptor 2
*GAPDH*: Glyceraldehyde-3-phosphate dehydrogenase
OE: Ovarian endometrioma
*PGR*: Progesterone receptor
qRT-PCR: Quantitative real-time RT-PCR
T-DMR: Tissue-dependent and differentially methylated region
